# Moral injury and psychological resilience among healthcare professionals amid COVID-19 pandemic

**DOI:** 10.12669/pjms.38.5.5122

**Published:** 2022

**Authors:** Mubeen Akhtar, Fayyaz Ahmad Faize, Ramla Zaid Malik, Asifa Tabusam

**Affiliations:** 1 Mubeen Akhtar, COMSATS University Islamabad (CUI), Park Road, Tarlai Kalan, Islamabad, Pakistan; 2 Fayyaz Ahmad Faize, COMSATS University Islamabad (CUI), Park Road, Tarlai Kalan, Islamabad, Pakistan; 3 Ramla Zaid Malik, COMSATS University Islamabad (CUI), Park Road, Tarlai Kalan, Islamabad, Pakistan; 4 Asifa Tabusam, COMSATS University Islamabad (CUI), Park Road, Tarlai Kalan, Islamabad, Pakistan

**Keywords:** Moral injury, Psychological resilience, COVID-19, Healthcare Professionals

## Abstract

**Objectives:**

The present research studied moral injury and psychological resilience in healthcare professionals amid COVID-19 pandemic. Relationship between moral injury and resilience was explored in addition to finding the difference in study variables based on socio-demographics factors.

**Methods:**

This cross-sectional research was carried out from August 2020 to January 2021. A sample of 108 healthcare professionals including doctors, nurses, and paramedical staff was collected through purposive sampling technique. Data was gathered through face-to-face survey method and online forum using psychometrically sound tools.**
**

**Results:**

Findings revealed that more than two third of the sample (69.44%) has high level of moral injury which is clinically significant while only 30.56% fall within normal range. Moral injury has significant positive correlation with number of work hours (*p* < .05) whereas negative correlation with resilience (*p* < .01) and years of experience (*p* < .05). Women and health care professionals belonging to psychiatry department have reported to experience significantly high level of moral injury (*p* < .01).

**Conclusion:**

The findings of the study are helpful for stakeholders of health care system to better understand and prepare for the situations that brings moral injury and challenge psychological resilience particularly in times of pandemic, humanitarian crisis, or natural disasters.

## INTRODUCTION

COVID-19 has certainly revolutionized the way of life for millions across the globe. Healthcare workers in particular have faced the brunt of the pandemic and its effects; long working hours as well as worry due to direct exposure to the lethal virus has increased distress among healthcare professionals. This distress could manifest in the form of a number of psychological problems such as anxiety and depression. This study focus specifically on the moral injury that healthcare professionals may have potentially experienced during this pandemic.^1^

Though the typically used terms burnout^2^ and compassion fatigue^3^ are also characterized by feelings of helplessness, emotional exhaustion and confusion, moral injury in particular, occurs as a result of being subjected to difficult situations that may go against the clinician’s own personal morals.^4^ Healthcare professionals are not only constantly exposed to the suffering of other people, which in itself causes substantial psychological distress and compassion fatigue, they often find themselves in morally grey situations where they must make life-or-death decisions for their patients. In the current context of the COVID-19 pandemic, healthcare workers have had to worry about managing with the limited resources available to them. For those in direct contact with COVID-19 patients, ensuring the isolation of their patients from other individuals along with the ever-looming possibility of becoming carriers of the virus themselves are both potential causes of distress for healthcare workers. Healthcare workers may experience rage, guilt and distrust as well as self-blame, low self-esteem, harsh self-disciplining and feelings of uselessness and vulnerability.^5^ Moral injury happens because they are frustrated and cannot provide the care they are trained for and promised to deliver. Furthermore, moral injury has been found to lead to decreased empathy and difficulties in coping with occupational stress^6^ – both of which may be particularly harmful for healthcare professionals.

Another key concept that will be discussed in this paper is psychological resilience - an individual’s ability to withstand setbacks and bounce back positively from difficulties.^7^ Due to the nature of their work, resilience is undoubtedly an important quality for healthcare professionals to possess, but the need for resilient healthcare workers has only increased with COVID-19. The concept of resilience may also be expanded to include ‘health systems resilience’ - referring to the strength of the entire healthcare system in dealing with stressful situations and responding adequately to stressors,^8^ such as the COVID-19 pandemic.

### Rationale of the Study:

Most of the research concerning moral injury was carried out with army personnel and war trauma survivors. However, this is just a very recent development that moral injury among physicians and other health professionals has attracted attention in the mainstream literature. Within this scenario, current study particularly focused on pandemic perspective while exploring moral injury experiences of health professionals who are working in different capacities including doctors, nurses and paramedical staff. It also intends to explore the association between moral injury and psychological resilience along with investigating differences based on socio-demographic factors.

## METHODS

A cross-sectional research design was employed and this research was carried out from August 2020 to January 2021 after the ethical approval from departmental Ethics Review Committee (ERC) COMSATS University, Islamabad (Ref.# CUI-ISB/HUM/ERC-CPA/2020-041). The sample consisted of 108 participants, selected through purposive sampling technique, from a population of healthcare workers in government and private hospitals across Pakistan. The inclusion criterion for paramedics and nurses was a minimum experience of six months at a clinical setting. Since data was collected from different departments, a specialization degree was necessary for doctors for inclusion in the sample to make this a homogeneous group. The exclusion criterion was a history of three months or more of an extended break from practice for any reason during the past one year. The sample’s ages ranged from 20 to 51 years (*M*=27.67, *SD*=6.17) including both male and female participants. The detailed demographic characteristic of the sample is presented in Table-I.

**Table I:** Demographic Characteristics of the Sample and Group Differences on Moral Injury and Psychological Resilience (N=108).

Variables	Category	*f*	*%*	*Moral injury*				*Psychological resilience*			

				*M*	*SD*	*t/F*	*P*	*M*	*SD*	*t/F*	*p*
Gender	Male	45	41.7	39.97	10.90	3.21	.002	26.82	7.21	.041	.96
	Female	63	58.3	45.77	7.86			26.76	7.80		
Marital status	Single	78	72.2	43.05	9.70	.537	.593	26.19	7.31	1.37	.187
	Married	30	27.8	44.16	9.58			28.33	7.99		
Hospital type	Government	91	84.3	43.03	9.57	.817	.416	25.98	7.82	2.65	.009
	Private	17	15.7	45.11	10.14			31.12	3.38		
Profession	Doctor	56	51.9	41.69	10.67	2.17	.119	25.21	6.88	2.88	.061
	Nurse	29	26.9	46.20	6.94			27.83	7.03		
	Paramedics	23	21.3	43.82	9.39			29.30	8.94		
Specialty/department	Psychiatry	10	3.7	49.01	10.70	6.40	.000	33.75	5.67	1.23	.295
	Neurology	13	12.0	37.61	6.77			24.92	8.27		
	Cardiology	13	12.0	40.92	10.07			25.00	5.81		
	Gastro	9	8.3	30.88	6.86			23.67	9.51		
	Medical laboratory	7	6.5	41.57	6.75			28.14	10.74		
	Nursing	29	26.9	46.20	6.94			27.83	7.03		
	Others	27	30.6	46.57	9.48			27.03	6.90		
Work area/ duty station	OPD	39	36.1	45.69	9.58	1.74	.163	27.38	7.98	4.08	.009
	Inpatient ward	18	16.7	42.33	8.67			21.61	5.33		
	Emergency room	15	13.9	44.69	8.49			26.67	9.64		
	More than one	36	33.3	40.86	10.27			28.78	5.90		

### Moral Injury Symptoms Scale – Health Professionals (MISS-HP):

The ten-item scale assesses symptoms of moral injury including guilt, shame, moral concerns, loss of trust, loss of meaning, self-condemnation, and religious struggles. The questionnaire uses a 10-point Likert scale, with total scores ranging from 0-100 (the higher the score, the greater the moral injury). The scale has an established reliability (Cronbach’s alpha: a= 0.70) and validity and has been previously used as a screening instrument for moral injury among healthcare professionals during the COVID-19 pandemic. The cutoff score on the MISS-HP for identifying HPs with clinically significant MI symptoms was 36 or higher.^9^

### Connor-Davidson Resilience Scale (CD-RISC-10):

For measurement of psychological resilience, the Connor-Davidson Resilience Scale-10 was used.^10^ This scale contains 10 items corresponding to flexibility, self-efficacy, ability to regulate emotion, optimism and maintaining attention under stress. It is a 5-point Likert-type cumulative instrument (0 = never to 4 = almost always). A summation of the response to each scale’s item yields a score that ranges from a minimum of 0 to a maximum of 40 that signifies the highest level of resilience. This scale has excellent psychometric properties with a Cronbach’s alpha coefficient of 0.89 and correlation coefficient of 0.87.

A demographic data sheet was used to collect information regarding gender, age, marital status, designation, years of practice, specialization, hospital type, and number of hours at work in a week. Hospitals were contacted to seek their permission to obtain information from their employees. Data was collected through physical as well as online mode. Written consent was taken from all the participants and their participation was completely voluntary. T-tests, one-way ANOVA and correlation were computed using SPSS-21.

## RESULTS

Results presented in Table-II revealed that 69.44% of the sample has high level of moral injury while 30.56% reported to experience low level of moral injury symptoms.

**Table II:** Level of Moral Injury among Healthcare Professionals (N=108).

Level of moral injury	Corresponding score	Frequency	(%)
Low	Score below 36	33	30.56
High	Score 36 or above	75	69.44

Cronbach’s Alpha coefficient for Moral Injury Scale and Resilience Scale is shown in Table-III. The scales have very good reliability ranging from 0.81 to 0.86. Correlations between study variables are also presented in Table-III. Psychological resilience has significant negative correlation with moral injury (*p* < 0.01) whereas significant positive correlation with age and number of years of experience (*p* < 0.01). Moral injury has significant positive correlation with number of work hours (*p* < 0.05) whereas negative correlation with years of experience (*p* < 0.05).

**Table III:** Descriptive Statistics and Correlations between Study Variables (N=108).

	Variable	*a* (no. of items)	*M*	*SD*	1	2	3	4	5
1	Psychological resilience	.86 (10)	26.79	7.53	-	-.26**	.28**	.35**	.041
2	Moral injury	.81 (10)	43.36	9.64		-	.067	-.15*	.17*
3	Age	-	27.67	6.17			-	.88**	-.110
4	Experience	-	4.84	5.88				-	.040
5	Work hours	-	7.89	1.86					-

* p< .05;

** p< .01.

Findings presented in Table-I shows that there are significant differences in the level of moral injury with reference to gender and department of health care professionals. Women have significantly high level of moral injury as compared to men (*p* < 0.01). Health care professionals belonging to psychiatry department have reported to experience significantly greater level of moral injury as compared to professionals working in other departments (*p* < 0.01).

Table-I also shows that there is significant difference in psychological resilience of participants based on the hospital type and duty station. Healthcare professionals in private hospitals have high resilience as compared to government hospital employees (*p* < 0.01). Individuals having duty in more than one station and participants working in OPD have high resilience as compared to participants in emergency rooms and inpatient wards (*p*< 0.01).

## DISCUSSION

More than two third of the sample (69.44%) was found to experience clinically significant moral injury symptoms during the time of pandemic. Mean item analysis showed that feeling guilt over failing to save someone from being seriously injured/dying, being troubled by having acted in ways that violated one’s own morals/values and feeling ashamed about what one done or not done when providing care to one’s patients were the topmost serious concerns for health professionals. A closer look at the differences in health professionals working in different roles suggest that there is no significant variation in the moral injury level of doctors, nurses and paramedical staff which imply they are all equally struggling with the constraints, lack of supplies and staff, and other pandemic shortfalls. Failing to consistently meet patients’ needs has a profound impact on their wellbeing which makes the basis of ensuing moral injury.^11^

There is a significant negative correlation between moral injury and resilience, suggesting that while one increases the other decreases. This finding is in accord with previous literature, where it is apparent that moral injury and psychological resilience may be regarded as two opposite ends of a pole.^12^ The very definition of moral injury involves feelings of guilt and shame when faced with emotionally and morally taxing situations. Such an experience is certainly at par with psychological resilience – which a person displays as an expression of strength in the face of adversity, and then positively adapts to that situation.^13^

A further look at the correlations of moral injury and psychological resilience with other variables reveals that resilience was found to be higher among senior healthcare professionals (both in age and in years of experience). This finding may be attributed to the advantage that senior practitioners have over their juniors in terms of experience and training.^14^ Greater exposure to stressful conditions may help develop resilience among the seniors and they may also learn specific coping strategies which reduce the level of stress they experience.^15^ Furthermore, senior professionals may also have greater confidence in themselves and their medical knowledge due to their many years of work^15^, thus reducing the guilt and low self-esteem that is observed in moral injury.

Moral injury, on the other hand is positively correlated to the number of work hours of the professional. This finding too can be explained by the fact that professionals who are overburdened with long working hours are not only exposed to more patients and potentially stressful cases but may also be occupied by thoughts of being unable to complete their work or performing inadequately while on duty. Moral injury was also found to be higher in professionals working in the psychiatry department. This could be the case due to the nature of psychiatric illnesses, which often cannot be outwardly observed or treated. Psychiatric cases are often complex and require professionals to invest themselves deeply into the case, sometimes even triggering their own personal vulnerabilities.^3^ Absorbing and then carrying around so much emotional baggage may certainly be a draining job for psychiatrists, which may eventually lead to moral injury.

Current study found women health care professionals to have significantly high level of moral injury as compared to men. However, no significant difference is seen concerning psychological resilience. This finding corroborates with past research which suggest a greater vulnerability of women to be diagnosed with anxiety, depression, and PTSD after being exposed to traumatic experience.^16^ Pertaining to marital status, profession, duty station, and hospital type, no significant group differences in moral injury were found.

An interesting finding that emerged from this study include the fact there was greater resilience found among professionals working at private hospitals rather than government hospitals. This result is surprising because private hospitals in Pakistan tended to be less crowded than government hospitals, where most patients visit due to their minimal fees. It could be surmised that professionals at government hospitals would be more resilient due to their long working hours and stressful environment since they could potentially learn coping strategies when constantly faced with such situations, however, the results suggest differently mainly because less patients means less moral injury which is then inversely related to resilience.

### Limitations of the Study:

The findings from the current study are specific in several aspects that may impact their interpretation. Due to pandemic, the accessibility of data from private hospital was difficult, therefore most of the data was taken from government hospitals. Majority of the sample was doctors (51%), which may limit the generalizability of findings to nurses and paramedics.

## CONCLUSION

Finding of this study revealed that healthcare professionals experienced significant level of moral injury during COVID-19 outbreak. Moral injury is inversely related to psychological resilience and thus taxing wellbeing. Further research is needed to design interventions and suggest coping strategies to improve overall conditions in healthcare system.

### Authors Contribution

**MA** conceived, designed and did statistical analysis & editing of manuscript.

**RZM and AT** did data collection and manuscript writing.

**FAF** has significant input in data analysis and revising article critically for important intellectual content.

**MA** takes the responsibility and is accountable for all aspects of the work in ensuring that questions related to the accuracy or integrity of any part of the work are appropriately investigated and resolved.
